# Effect of Annealing Temperature for Ni/AlO_x_/Pt RRAM Devices Fabricated with Solution-Based Dielectric

**DOI:** 10.3390/mi10070446

**Published:** 2019-07-02

**Authors:** Zongjie Shen, Yanfei Qi, Ivona Z. Mitrovic, Cezhou Zhao, Steve Hall, Li Yang, Tian Luo, Yanbo Huang, Chun Zhao

**Affiliations:** 1 Department of Electrical and Electronic Engineering, Xi’an Jiaotong-Liverpool University, Suzhou 215123, China; 2 Department of Electrical Engineering and Electronics, University of Liverpool, Liverpool L69 3BX, UK; 3 School of Electronic and Information Engineering, Xi’an Jiaotong University, Xi’an 710061, China; 4 Department of Chemistry, Xi’an Jiaotong-Liverpool University, Suzhou 215123, China; 5 Department of Chemistry, University of Liverpool, Liverpool L69 3BX, UK

**Keywords:** bipolar resistive switching characteristics, annealing temperatures, solution-based dielectric, resistive random access memory (RRAM)

## Abstract

Resistive random access memory (RRAM) devices with Ni/AlO_x_/Pt-structure were manufactured by deposition of a solution-based aluminum oxide (AlO_x_) dielectric layer which was subsequently annealed at temperatures from 200 °C to 300 °C, in increments of 25 °C. The devices displayed typical bipolar resistive switching characteristics. Investigations were carried out on the effect of different annealing temperatures for associated RRAM devices to show that performance was correlated with changes of hydroxyl group concentration in the AlO_x_ thin films. The annealing temperature of 250 °C was found to be optimal for the dielectric layer, exhibiting superior performance of the RRAM devices with the lowest operation voltage (<1.5 V), the highest ON/OFF ratio (>10^4^), the narrowest resistance distribution, the longest retention time (>10^4^ s) and the most endurance cycles (>150).

## 1. Introduction

As one of the promising candidates for next-generation nonvolatile memories, resistive random access memory (RRAM) has received considerable attention due to significant advantages concerning simplicity of structure, low power consumption, fast read & write speed, high scalability and 3-D integration feasibility compared to the industry standard silicon-based flash memories [1,2,3,4,5,6,7]. Current candidate materials for the resistive switching (RS) layer of RRAM devices include perovskite, ferromagnetic and metal oxide-based materials [1,3,4,5,8,9,10,11]. In particular, metal oxide-based materials such as AlO_x_, NiO_x_, TiO_x_ and HfO_x_ are currently extensively discussed because of the simplicity of the material [10,12,13,14]. Among these materials, AlO_x_ has been widely applied in gate insulator layers [15,16,17,18] and has attracted extensive attention in the RRAM field owing to its wide band gap (~8.9 eV), high thermal stability with Si and Pt, high dielectric constant (~8) and large breakdown electric field [10,14,19,20,21,22] as Kim et al. has reported [19,20,23,24,25,26]. In addition, the superior elasticity [27] and high toughness [28] make it possible for AlO_x_ to be applied under various conditions including vibration and pressure environments [29,30,31]. Cano et al. reported that AlO_x_-based dielectric layer showed superior stability under environments with hydrofluoric acid pressure [29] and Choi et al. reported large-scale flexible electronics application with AlO_x_ thin film [31], which have demonstrated that the AlO_x_ thin film has great potential as a metal oxide layer in RRAM devices. 

A number of fabrication methods for incorporation of a metal oxide RS layer in AlO_x_-based RRAM devices have been investigated. Methods based on solution processes for metal oxide thin films have been extensively considered, namely spin [32,33,34] and dip coating [35,36,37], drop casting [34,36,37,38] and different printing methods. Compared with traditional fabrication methods such as atomic-layer-deposition (ALD) [17,39,40] and magnetron sputtering [28,40,41], the solution-based method has advantages of low fabrication cost with the elimination of vacuum deposition processes [42], ease of preparation for precursor materials [39,43,44] and high efficiency of device throughput [27], which reveals the promising prospect of solution-based methods in RS layer fabrication. Several factors including plasma cleaning time, deposition gaseous environment and annealing temperature are considered to influence the performance of solution-based metal oxide thin films. A limited number of investigations have been reported regarding the relationship between annealing temperature and performance of RRAM device with solution-based RS layer [10,38].

In this work, the AlO_x_ thin film was deposited with a spin-coating method and then annealed at temperatures of 200 °C to 300 °C, in increments of 25 °C. The RRAM devices with solution-based AlO_x_ thin film were characterized electrically in terms of operation voltage, ON/OFF ratio between the high resistance state (HRS) and low resistance state (LRS), resistance distribution, retention time and endurance cycles. X-ray photoelectron spectroscopy (XPS) results indicate that these performance metrics are associated with different gradients of hydroxyl group (-OH) concentrations in the AlO_x_ thin films with different annealing temperatures. Devices with AlO_x_ thin films annealed at 250 °C demonstrated superior performance with the lowest operation voltage (<1.5 V), the highest ON/OFF ratio (>10^4^), the narrowest resistance distribution, the longest retention time (>10^4^ s) and the most endurance cycles (>150).

## 2. Device Fabrication 

**Figure 1:**
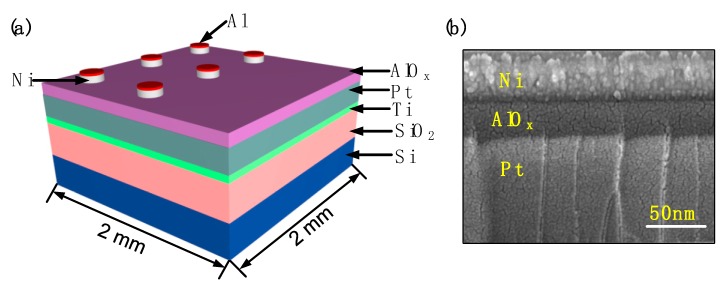
(**a**) Schematic of an Al/Ni/solution-based AlO_x_/Pt RRAM device; (**b**) a scanning electron microscope (SEM) cross-sectional image of the Al/Ni/solution-based AlO_x_/Pt RRAM device.

The fabricated Ni(top)/AlO_x_/Pt(bottom) memory device structure with dimensions 2 mm × 2 mm is shown in Figure 1a. Firstly, the substrate comprising layers Pt (200 nm)/Ti/SiO_2_/Si was ultrasonically cleaned in acetone, ethanol and deionized (DI) water, sequentially. Then an aluminum nitrate nonahydrate (Al(NO_3_)_3_·9H_2_O) solution consisting of ~9.353 g Al(NO_3_)_3_·9H_2_O and 10 mL deionized water was prepared as the 2.5 M AlO_x_ precursor. The precursor solution was stirred vigorously for 20 min under ambient air conditions. The Pt substrate surface layer was given a hydrophilic treatment in a plasma cleaner in an atmospheric environment. The AlO_x_ precursor solution, filtered through a 0.45 μm polyether sulfone (PES) syringe, was spin-coated onto the substrate at a spin rate of 4500 rpm for 40 s and subsequently annealed at the different desired temperatures of 200 °C, 225 °C, 250 °C, 275 °C and 300 °C for 60 min under ambient conditions. A ~40 nm-thick top electrode (TE) layer of Ni and a ~40 nm-thick capping layer of Al were both deposited by e-beam evaporation. Figure 1b shows a scanning electron microscope (SEM) cross-sectional image of the device, confirming the target thicknesses of ~40 nm, ~30 nm and ~100 nm for Ni, AlO_x_ and Pt layers respectively. 

An Agilent B1500A high-precision semiconductor analyzer (Agilent Santa Rosa, CA, USA) was employed to measure the I-V characteristics with a two-probe configuration. All electrical measurements were performed in the dark and at room temperature within a Faraday cage. In addition, to investigate the effect of annealing temperatures on device performance, X-ray photoelectron spectroscopy (XPS) spectra of constituent Al and O core level (CL) elements were measured.

## 3. Results and Discussion

### 3.1. Memoristic Characteristics Based on Al/Ni/Solution-Based AlO_x_/Pt RRAM

**Figure 2:**
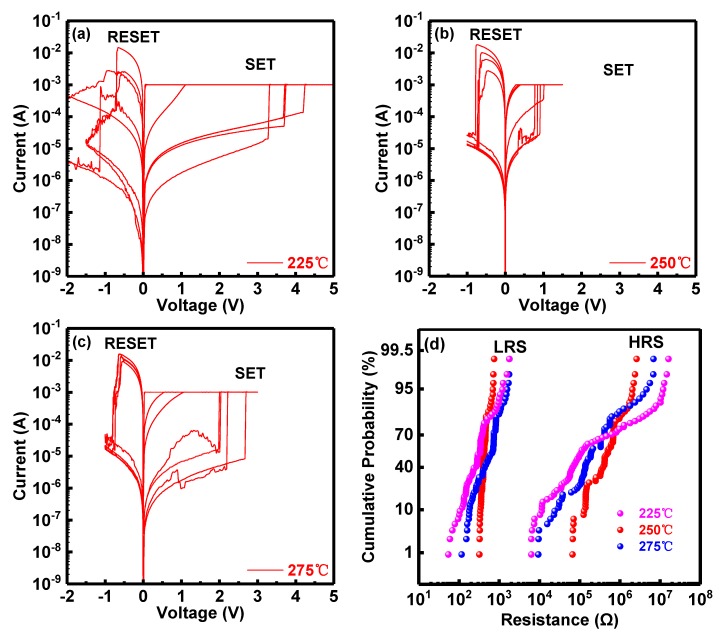
I-V curves of Al/Ni/solution-based AlO_x_/Pt RRAM devices with (resistive switching) RS layer annealed at (**a**) 225 °C; (**b**) 250 °C and (**c**) 275 °C. (**d**) Resistance distribution of Al/Ni/solution-based AlO_x_/Pt RRAM device with RS layer deposited at various temperatures.

**Figure 3:**
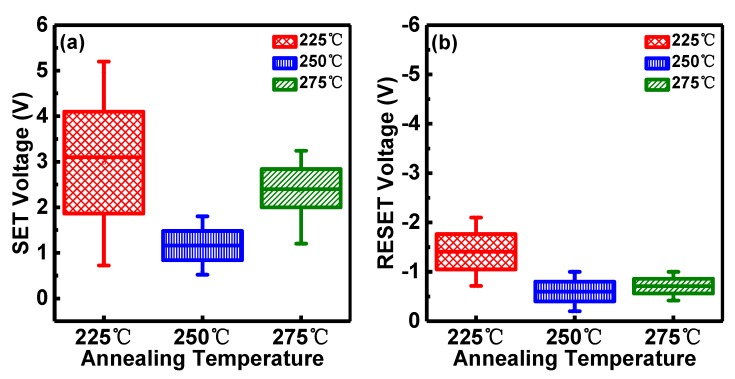
Voltage distribution of (**a**) SET operation and (**b**) RESET operation for Al/Ni/solution-based AlO_x_/Pt RRAM devices with RS layer annealed at different temperatures.

The RRAM devices were operated under 1 mA compliance current (CC) and observed to exhibit typical bipolar RS behavior, as illustrated by the I-V characteristics in Figure 2. The devices with the dielectric layer annealed at 200 °C exhibit typical RRAM breakdown characteristics at very low voltage <0.3 V while breakdown characteristics of 300 °C annealed devices are not usually observed even for voltages higher than 18 V, which is of course, unsuitable for RRAM device application [45,46]. Therefore, RRAM devices with dielectric layers annealed at 225 °C, 250 °C, 275 °C were considered for further evaluation. Compared with unipolar I-V characteristics of other RRAM devices [47], all RRAM devices with Al/Ni/solution-based AlO_x_/Pt structure demonstrate typical bipolar I-V characteristics without forming operation. The current compliance (CC) is set at 1 mA to prevent catastrophic breakdown of the RRAM devices. During cycling, the HRS was transferred to LRS abruptly in the SET process and the resistance of the LRS began to increase abruptly toward HRS in the RESET process. The SET and RESET process controls the RRAM device transition to ON and OFF states. It is observed that the majority of values of SET voltages (V_SET_) for the three samples are around 1.5 V while some are up to 4 V. In the RESET process, nearly all RESET voltages (V_RESET_) are around −1 V approximately. As illustrated in Figure 3, in the SET operation, the average values of V_SET_ are around 3.2 V, 1.0 V and 2.4 V at 225 °C, 250 °C and 275 °C, respectively. RRAM devices with dielectric layer annealed at 250 °C exhibit the lowest SET voltages (Figure 3a) with the highest ON/OFF ratio (>10^4^) between LRS (ON state) and HRS (OFF state). Similar results can be observed in the RESET operation (Figure 3b) although the variation of V_RESET_ average values is not as obvious as that of V_SET._ Figure 2d shows the cumulative probability for resistance distribution of the RRAM devices annealed at various temperatures. All values of memory resistance at HRS (R_HRS_) and LRS (R_LRS_) of consecutive forming-free DC switching cycles were read at 0.1 V. As illustrated in Figure 2d, curves of resistance distribution almost overlap at LRS, indicating that no significant dependence on annealing temperature is apparent at LRS. However, an obvious variation can be observed at R_HRS_. The uniformity and narrowness of the resistance distribution are key metrics for stability and quality of RRAM devices. A narrow resistance distribution is considered to be a good demonstration of the stability and performance of devices [7,48,49,50]. In this work, the narrowest resistance distribution of Al/Ni/solution-based AlO_x_/Pt RRAM devices is found for the 250 °C annealing temperature, which therefore presents the best uniformity of the devices.

### 3.2. Endurance and Retention Properties of Al/Ni/Solution-Based AlO_x_/Pt RRAM

**Figure 4:**
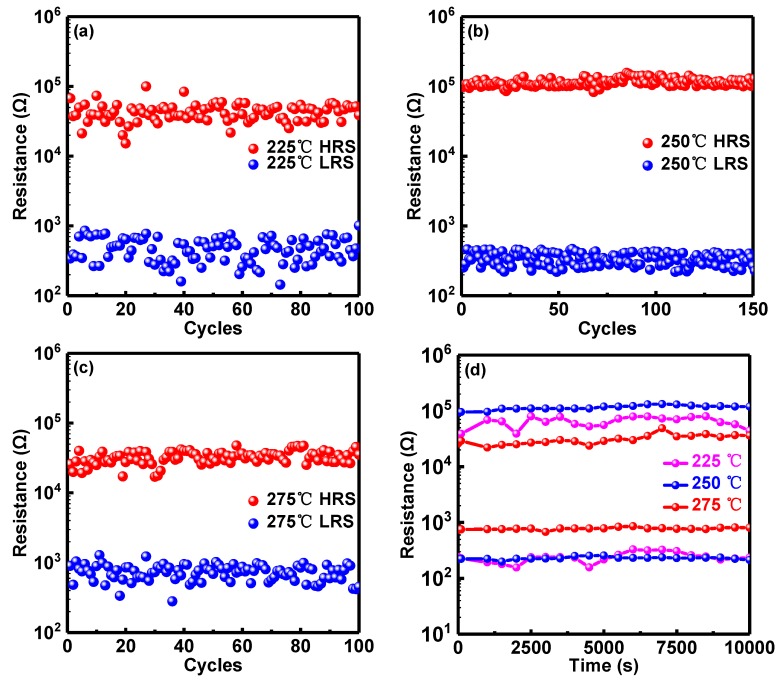
Endurance property of Al/Ni/solution-based AlO_x_/Pt RRAM devices with RS layer annealed at (**a**) 225 °C; (**b**) 250 °C and (**c**) 275 °C. (**d**) Retention property of RRAM devices annealed at various temperatures.

Figure 4 demonstrates the retention and endurance properties at HRS and LRS for the RRAM devices with RS layers annealed at various temperatures. With the results of resistance distribution above, the resistance values of retention and endurance belong to the range of HRS and LRS values in Figure 2d. Resistance values both at HRS and LRS are read at 0.2 V. Figure 4a–c show DC cycles vs resistance at 1 mA CC of devices annealed at 225 °C, 250 °C and 275 °C, which show similar characteristics to those observed in the resistance distribution of Figure 2d. The best resistance distribution can be observed in 250 °C annealed RRAM devices and the worst uniformity of resistance can be observed in 225 °C annealed RRAM devices. Similarly, the endurance property with the best uniformity is demonstrated in the RRAM device annealed at 250 °C while the worst performance is observed in the RRAM device annealed at 225 °C. The same retention property can be observed in Figure 4d, which shows that the device can sustain data for more than 10^4^ s. 

The best performance was found for an annealing temperature of 250 °C with the lowest operation voltage (<1.5 V), the highest ON/OFF ratio (>10^4^), the narrowest resistance distribution, the longest retention time (>10^4^ s) and the most endurance cycles (150).

### 3.3. Switching Mechanism of Al/Ni/Solution-Based AlO_x_/Pt RRAM

**Figure 5:**
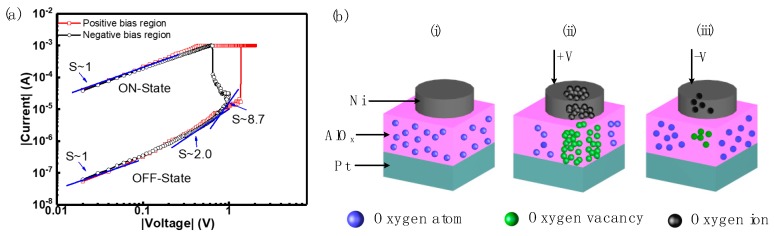
(**a**) Curve fitting of I-V characteristics for Al/Ni/solution-based AlO_x_/Pt RRAM devices indicating SCLC conduction. (**b**) Diagrams to describe the switching mechanism of Al/Ni/solution-based AlO_x_/Pt RRAM devices at (i) the initial state, (ii) the ON state and (iii) the OFF state, respectively.

With typical bipolar RS performance demonstrated by Al/Ni/solution-based AlO_x_/Pt RRAM devices, the RS modeling with fitting curves (250 °C annealed devices) illustrated in Figure 5a is used to investigate the conduction mechanism. Figure 5a shows evidence for space-charge limited current (SCLC) as the dominant conduction mechanism in 250 °C annealed devices. The fitting results show positive and negative bias regions of I-V characteristics in double logarithmic plots. A large area overlap of SET and RESET can be observed due to the approximately equal values of CC and RESET current. The currents are seen to follow Ohmic conduction (I ∝ V) in the low voltage regime [51,52]. At higher bias voltages, the OFF-state slope shows a transition to about 2.0, consistent with Child’ s square law [53,54]. By further increasing the applied voltage, the slope increased to approximately 8.7, again consistent with the SCLC mechanism [53,54,55,56].

Bipolar RS performance of all RRAM devices with different annealing temperatures are considered to be associated with the formation and rupture process of conductive filaments (CF) associated with oxygen vacancies, in the SET/RESET process [2,3,4,15,57]. Figure 5b shows a schematic representation of this process consisting of ON and OFF states, which is considered as the switching mechanism of these devices. The formation and rupture process of CF is associated with the distribution of oxygen ions and oxygen vacancies in the TE and RS layer [22,48,57,58,59]. Figure 5b(i) shows the initial state of RRAM devices without applied voltage, indicating oxygen atoms present in the AlO_x_ thin film. With application of a positive voltage to the Ni electrode in the SET operation, electrons are captured by oxygen atoms in the AlO_x_ thin film [15,27,60,61,62], to yield oxygen ions which drift to TE. The generation process of oxygen ions can be represented as:

$$O+\;2e^{-}\;\to \;O^{2-}$$

The oxygen vacancies remain in the AlO_x_ thin film and constitute the dominant components of CF. This formation process of CF consisting of oxygen vacancies in the AlO_x_ thin film is considered to be responsible for the resistance state transition (HRS to LRS) of RRAM devices at the ON state, as depicted in Figure 5b [48,58,60]. Conversely, in the RESET operation, with a negative voltage applied to TE, oxygen ions stored in the electrode drift back to the AlO_x_ thin film under the influence of the negative electrical field and therefore reduce the density of oxygen vacancies in the AlO_x_ thin film [48,63]. This action dominates the rupture process of CF [15,22,48] and the RRAM devices perform at the OFF state (LRS to HRS).

The formation and rupture mechanism of CF is confirmed to be associated with the characteristics of the RS layer in filamentary RRAM devices with the dependency on film thickness, measurement temperature and deposition temperature [64,65,66,67]. In this work, the device performance is found to be dependent on annealing temperature of the dielectric layer and the best performance is observed in the device with a dielectric layer annealed at 250 °C.

**Figure 6:**
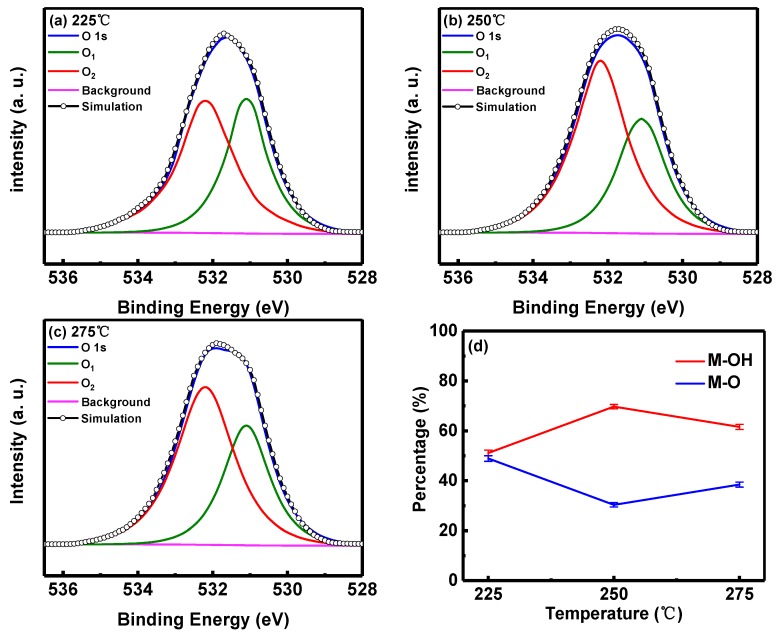
XPS spectra of O 1s CLs for Al/Ni/solution-based AlO_x_/Pt RRAM devices annealed at (**a**) 225 °C; (**b**) 250 °C and (**c**) 275 °C. (**d**) Integrated intensities of O 1s CL sub-peak referring to M-OH bond and M-O bond for solution-based AlO_x_ layers annealed at different temperatures.

Physical characterization was undertaken using XPS. Figure 6a–c show XPS spectra of O 1s core levels for the AlO_x_ thin films annealed at 225 °C, 250 °C and 275 °C. The O 1s CL spectrum can be de-convoluted into two sub-peaks with binding energies located at 531.1 eV (O_1_) and 532.2 eV (O_2_) [40,64,65,66,67]. The O_1_ and O_2_ peaks are associated with the metal-oxygen bonds (O_1_) and hydroxyl group (O_2_), respectively [5,66,67]. As illustrated in Figure 6a–c, the hydroxyl-related peak (O_2_) increased with annealing temperatures from 225 °C to 250 °C and decreased from 250 °C to 275 °C. Similar behavior has been observed by Xu et al. [68]. The highest and the lowest concentration of the hydroxyl group is found for samples annealed at 250 °C and 225 °C, respectively. Figure 6d shows the integrated intensity of the two sub-peaks referring to the concentration of hydroxyl group (M-OH) and metal-oxygen bonds (M-O) for the three samples. The observed variation in concentration of hydroxyl group has been found to show strong correlation to RRAM device performance. The best performing RRAM device annealed at 250 °C has the highest concentration of hydroxyl group, while the worst performance is observed for device annealed at 225 °C which exhibits the lowest concentration of hydroxyl group.

With the different concentrations of M-O and M-OH in the dielectric layer, two main species of compositions, namely AlO_x_ and Al(OH)_x_, play dominant roles in switching behavior. We now propose a hypothesis for the relationship between composition and surface roughness of the dielectric layer. The more complex the compositions of the dielectric layer, the higher surface roughness will be present [69,70,71]. The surface roughness assessed by Atomic Force Microscope (AFM) of dielectric layers annealed at 225 °C, 250 °C and 275 °C are 0.682 nm, 0.230 nm and 0.524 nm, respectively. In 225 °C annealed devices, the similar concentration (~50%) of M-O and M-OH can be detected in the film indicating that the concentration of AlO_x_ and Al(OH)_x_ are almost equal. Hence the dielectric layer performance might be affected concurrently by two main compositions. A smooth surface of the dielectric layer is essential to achieve low leakage current and the realization of high-performance dielectric thin films. A higher concentration of M-OH is observed in the 250 °C annealed AlO_x_ thin film, which indicates that Al(OH)_x_ has a more dominant influence on the layer properties. Compared with Al(OH)_x_, the influence of AlO_x_ is less significant, which results in a lower surface roughness. In addition, the existence of the hydroxyl group in the dielectric layer is associated with water absorption, which affects the permittivity of AlO_x_ with a slight fluctuation (~9.3–~11.5) and hence the capacitance associated with the dielectric thin film. This part will be submitted to further investigation.

## 4. Conclusions

RRAM devices with Al/Ni/AlO_x_/Pt structure were fabricated by a solution-based process with the RS layer annealed at 200 °C, 225 °C, 250 °C, 275 °C and 300 °C. The effect on RRAM device performance for annealing temperatures of 225 °C, 250 °C, 275 °C was investigated in terms of the operation voltages of RS characteristics, resistance distribution, endurance cycles and retention uniformity. The worst device performance was observed for an annealing temperature of 225 °C and the better performance was demonstrated in the device annealed at 275 °C. The best performance was found for an annealing temperature of 250 °C with the lowest operation voltage (<1.5 V), the highest ON/OFF ratio (>10^4^), the narrowest resistance distribution, the longest retention time (>10^4^ s) and the most endurance cycles (150), which indicates the lowest energy consumption and the excellent stability of the RRAM devices. An XPS study has been conducted to determine elements present in the AlO_x_ thin films prepared at different annealing temperature with the aim of explaining the variation of associated RRAM devices performance. The device performance was considered to be related to the concentration gradient of hydroxyl groups in the solution-based AlO_x_ thin films for different annealing temperatures.
